# Supplementary material for: Voluntary Medical Male Circumcision’s (VMMC) strategy for HIV prevention in Sub-Saharan Africa, prevalence, risks, costs, benefits and best practice: A scoping review study protocol of progress and unfolding insights
Source: PLoS One. 2024 Dec 31;19(12):e0316106. doi: 10.1371/journal.pone.0316106 (PMC11687801; doi:10.1371/journal.pone.0316106)
Supplement: S1 Checklist — (DOCX) [file pone.0316106.s001.docx]

**PRISMA-P (Preferred Reporting Items for Systematic review and Meta-Analysis Protocols) 2015 checklist: recommended items to address in a systematic review protocol***

| Section and topic | Item No | Checklist item | Description |
| --- | --- | --- | --- |
| ADMINISTRATIVE INFORMATION | | |  |
| Title: |  |  |  |
| Identification | 1a | Identify the report as a protocol of a systematic review | The protocol is for a scoping review |
| Update | 1b | If the protocol is for an update of a previous systematic review, identify as such | N/A |
| Registration | 2 | If registered, provide the name of the registry (such as PROSPERO) and registration number | It is registered with the OSF, https://doi.org/10.17605/OSF.IO/SFZC9 |
| Authors: |  |  |  |
| Contact | 3a | Provide name, institutional affiliation, e-mail address of all protocol authors; provide physical mailing address of corresponding author | - Charles Maibvise, University of Hertfordshire, [Cmaibvise08@yahoo.com](mailto:Cmaibvise08@yahoo.com) - Takaedza Munangatire*, University of Namibia, [takamunangatire@gmail.com](mailto:takamunangatire@gmail.com), [Maria Mwengere Road, Rundu, Namibia] - Nestor Tomas, University of Namibia, [tomas.npn@gmail.com](mailto:tomas.npn@gmail.com) - Daniel O. Ashipala, University of Namibia, [doashipala@gmail.com](mailto:doashipala@gmail.com), - Priscilla S. Dlamini, University of Eswatini, [Psdlamini@uniswa.sz](mailto:Psdlamini@uniswa.sz), |
| Contributions | 3b | Describe contributions of protocol authors and identify the guarantor of the review | All authors contributed in the conceptualisation of the paper and refinement of the write-up. They will also participant data collection and extraction, and refinement of the final report.  Takaedza Munangatire is the guarantor of the review |
| Amendments | 4 | If the protocol represents an amendment of a previously completed or published protocol, identify as such and list changes; otherwise, state plan for documenting important protocol amendments | N/A |
| Support: |  |  |  |
| Sources | 5a | Indicate sources of financial or other support for the review | Self-funded |
| Sponsor | 5b | Provide name for the review funder and/or sponsor | None |
| Role of sponsor or funder | 5c | Describe roles of funder(s), sponsor(s), and/or institution(s), if any, in developing the protocol | N/A |
| INTRODUCTION | | |  |
| Rationale | 6 | Describe the rationale for the review in the context of what is already known | There are still uncertainties regarding the risk benefit ratios of VMMC as an HIV prevention strategy, yet from the start of the campaigns over 10 years ago, enough evidence must have been generated to enlighten health practitioners about cost-benefits ratio. In addition, there are scattered and/or isolated reports of progress towards set VMMC targets in different places. This study aims at synthesising and consolidating those pieces of evidence and draw a conclusion that can help inform the future of the strategy. |
| Objectives | 7 | Provide an explicit statement of the question(s) the review will address with reference to participants, interventions, comparators, and outcomes (PICO) | The specific research questions:   - What is the prevalence of male circumcision in Sub-Sahara Africa? - What evidence has been generated regarding VMMC in terms of risks, costs, benefit and best practice in the performance of the procedure? |
| METHODS | | |  |
| Eligibility criteria | 8 | Specify the study characteristics (such as PICO, study design, setting, time frame) and report characteristics (such as years considered, language, publication status) to be used as criteria for eligibility for the review | The “PCC” framework was used, where the population (P) of interest are men and children aged ten (10) years or older, being the primary targets of VMMC at some point. The concept (C) under study is the VMMC strategy for HIV prevention, with a particular interest in gained insights that are worth considering in evaluating the cost-benefit ratio of VMMC. The Context (C) for the study is Sub-Saharan Africa, where the strategy was rolled out. |
| Information sources | 9 | Describe all intended information sources (such as electronic databases, contact with study authors, trial registers or other grey literature sources) with planned dates of coverage | Targeted data sources are: Web of Science, Cochrane Library, Scopus, Science Direct, PubMed as well as WHO Institutional Repository for Information Sharing (IRIS). |
| Search strategy | 10 | Present draft of search strategy to be used for at least one electronic database, including planned limits, such that it could be repeated | (“men” OR “man” OR “male”) AND (“VMMC” OR “circumcision”) AND (“benefits” OR “risks” OR “complications” OR “adverse effects” OR “cost” OR “prevalence” OR “progress”) |
| Study records: |  |  |  |
| Data management | 11a | Describe the mechanism(s) that will be used to manage records and data throughout the review | Passworded Microsoft OneDrive cloud storage spaces shall be use, with links shared among authors only. |
| Selection process | 11b | State the process that will be used for selecting studies (such as two independent reviewers) through each phase of the review (that is, screening, eligibility and inclusion in meta-analysis) | Mendeley referencing manager will be used, with passworded individual accounts |
| Data collection process | 11c | Describe planned method of extracting data from reports (such as piloting forms, done independently, in duplicate), any processes for obtaining and confirming data from investigators | A pre-designed data extraction template shall be used. |
| Data items | 12 | List and define all variables for which data will be sought (such as PICO items, funding sources), any pre-planned data assumptions and simplifications | Details of the article (Author (s), year of publication, location of the study), Title of article, Type of source (Peer reviewed journal, grey literature, international guidelines, report), Study design/method (Quantitative; Qualitative; Mixed method), Aim/purpose (Overall aim or objective of the study), target population and sample size, type of intervention (Details of intervention and/or comparator if applicable), Concept of VMMC covered or reported (e.g VMMC prevalence; risks; costs; complication; benefits; recommendations for best practice), Results/findings, as well as Additional information such as limitations of the study, validity and trustworthiness of findings/ |
| Outcomes and prioritization | 13 | List and define all outcomes for which data will be sought, including prioritization of main and additional outcomes, with rationale | The following shall be sought in relationship to the VMMC programme and the surgical procedure: Complications, Risks, Benefits, Costs, Recommendations and Coverage, or Prevalence, that is, Progress towards set targets. |
| Risk of bias in individual studies | 14 | Describe anticipated methods for assessing risk of bias of individual studies, including whether this will be done at the outcome or study level, or both; state how this information will be used in data synthesis | The Mixed Method Appraisal Tool (MMAT) for scoping review shall be used, and this shall be done at outcome level. |
| Data synthesis | 15a | Describe criteria under which study data will be quantitatively synthesised | Reported quantifications in reviewed literature shall also be captured as such in this review, and critically analysed to capture the connotation of the reported quantities. |
|  | 15b | If data are appropriate for quantitative synthesis, describe planned summary measures, methods of handling data and methods of combining data from studies, including any planned exploration of consistency (such as I^2^, Kendall’s τ) | N/A |
|  | 15c | Describe any proposed additional analyses (such as sensitivity or subgroup analyses, meta-regression) | None |
|  | 15d | If quantitative synthesis is not appropriate, describe the type of summary planned | An inductive qualitative summary of the findings will be developed. |
| Meta-bias(es) | 16 | Specify any planned assessment of meta-bias(es) (such as publication bias across studies, selective reporting within studies) | None is planned for this protocol. It is envisioned that findings of this review will form the basis of a systematic review where such further analysis will be performed. |
| Confidence in cumulative evidence | 17 | Describe how the strength of the body of evidence will be assessed (such as GRADE) | No further scrutiny shall be done in that regard, on assumption that the selected data bases publish relatively credible literature |

*** It is strongly recommended that this checklist be read in conjunction with the PRISMA-P Explanation and Elaboration (cite when available) for important clarification on the items. Amendments to a review protocol should be tracked and dated. The copyright for PRISMA-P (including checklist) is held by the PRISMA-P Group and is distributed under a Creative Commons Attribution Licence 4.0.**

*From: Shamseer L, Moher D, Clarke M, Ghersi D, Liberati A, Petticrew M, Shekelle P, Stewart L, PRISMA-P Group. Preferred reporting items for systematic review and meta-analysis protocols (PRISMA-P) 2015: elaboration and explanation. BMJ. 2015 Jan 2;349(jan02 1):g7647.*
